# Editorial: Approaches to, and the implications of, timing of birth

**DOI:** 10.3389/fgwh.2023.1244492

**Published:** 2023-08-11

**Authors:** Peter von Dadelszen, Corine J. M. Verhoeven, Wessel Ganzevoort

**Affiliations:** ^1^ Institute of Women and Children’s Health, King’s College London, London, United Kingdom; ^2^ Department of Midwifery Science, Amsterdam University Medical Centre, University of Amsterdam, Amsterdam, Netherlands; ^3^ Amsterdam Public Health Research Institute, Amsterdam, Netherlands; ^4^ Division of Midwifery, School of Health Sciences, University of Nottingham, Nottingham, United Kingdom; ^5^ Department of Obstetrics and Gynaecology, Maxima Medical Centre, Veldhoven, Netherlands; ^6^ Department of Obstetrics, Amsterdam University Medical Centre, University of Amsterdam, Amsterdam, Netherlands; ^7^ Amsterdam Reproduction and Development Research Institute, Amsterdam, Netherlands

**Keywords:** pregnancy, timing of birth, evidence-based medicine, induction of labour, elective caesarean birth

**Editorial on the Research Topic** Approaches to, and the implications of, timing of birth

In late 2022, we proposed a Research Topic entitled *Approaches to, and the implications of, timing of birth*. We are delighted to have supported the publication of four manuscripts within the topic (McLaughlin et al.; Molla et al.; Roba et al.; von Dadelszen et al.). To us, it is interesting to observe how various groups of investigators responded to the topic.

Our observation is that, for three of the manuscripts, there is an underlying common thread about gathering information to enable and enhance shared decision making about the timing and place of giving birth (McLaughlin et al.; Molla et al.; von Dadelszen et al.). While most women and their families desire to experience a spontaneous onset of labour, it is a reality that in some cases timed birth may be the better option if the goal is to avoid a Caesarean birth, while in other cases it might be better to wait for this spontaneous onset. The decision is never between labour induction and spontaneous onset of labour, it is between induction and ongoing pregnancy that may end with either spontaneous labour or a medically-indicated birth (i.e., induction or elective/semi-elective Caesarean birth)—when pregnancies are complicated by, say, an increased risk of hypertension, then the natural history is of a high rate of interventions proportional to the level of that developed risk (1). Indeed, in both uncomplicated and complicated pregnancies at term, in randomised controlled trials induction appears to consistently increase the opportunities to give birth vaginally (2–5), albeit that this may not be the only outcome of value to women and their context. It is important to notice that in observational studies, such as registry-based studies, it seems the other way around: in regions with low induction of labour rates, also fewer unplanned Caesareans were observed (6). This apparent contradiction requires ongoing investigation and is a source of debate amongst the editors.

With community engagement and feedback, McLaughlin and colleagues were able to introduce a programme of ultrasound and specialist consultation to guide the care of 500 pregnant Burundian women with previous Caesarean births. While overall Caesarean births increased, the rate of the more dangerous unscheduled Caesarean births decreased (McLaughlin et al.). Molla and colleagues describe their experience of caring for 264 Ethiopian women with ultrasound-detected oligohydramnios—a high rate of interventions ensued with an overall Caesarean birth rate of almost 60%; only two-thirds of the Caesarean births were elective (Molla et al.). This is important information to guide joint decision making and to create realistic expectations in pregnant women and their families. von Dadelszen and colleagues examined the relationship between the content of 21 induction of labour patient information leaflets and current evidence—the evidence-to-advice gap was substantial and almost universally biased against induction (von Dadelszen et al.). While celebrating the benefits of spontaneous labour and vaginal birth, we feel that maternity care providers have a responsibility to provide best evidence-based counselling and guidance to pregnant women and their families and not to bring unconscious biases to the counselling table.

The final paper by Roba and colleagues is an outlier in being focussed on the interactions between food insecurity and secondary subfertility assessed through accessing demographic and health surveys in 10 East African countries (Roba et al.). In addition to food insecurity and other factors, increased age at first birth was associated with subsequent subfertility.
